# Correlation between controlled ovarian stimulation protocols and euploid blastocyst rate in pre-implantation genetic testing for aneuploidy cycles

**DOI:** 10.1186/s12958-023-01166-7

**Published:** 2023-12-06

**Authors:** Bixia Huang, Hui Li, Bin Xu, Ning Li, Xiaofei Wang, Yanping Li, Jing Zhao

**Affiliations:** 1 Reproductive Medicine Center, Xiangya Hospital of Central South University, Changsha, China; 2 Clinical Research Center for Women’s Reproductive Health in Hunan Province, Changsha, Hunan China; 3 Reproductive Medicine Center, The Third Affiliated Hospital of Guangxi Medical University, Nanning, Guangxi China; 4 Reproductive Medicine Center, Chengdu Xinan Gynecology Hospital, Chengdu, Sichuan China

**Keywords:** Controlled ovarian stimulation, Euploid blastocyte, Pre-implantation genetic testing, Aneuploidy

## Abstract

**Background:**

Several studies have explored which COS protocol yields a higher blastocyst euploidy rate, but findings have been inconsistent. The present study aimed to explore whether controlled ovarian stimulation (COS) protocols was associated with euploid blastocyst rate in pre-implantation genetic testing for aneuploidy (PGT-A) cycles.

**Methods:**

The study was a retrospective study where data were obtained from three reproductive medicine centers. The study included PGT-A cycles with the GnRH-a, GnRH-ant, or PPOS protocols, and the data on patient demographics, protocols, and embryonic outcomes were collected for the PGT-A cycles performed between January 2019 and August 2022.

**Results:**

This study included 457 PGT-A cycles from three reproductive medicine centers, with 152, 126, and 179 cycles performed using the PPOS, GnRH-a, and GnRH-ant protocols, respectively. The baseline characteristics of the three groups show no significant differences were observed in female BMI, infertility type, and infertility duration among the PPOS, GnRH-a, and GnRH-ant protocol groups. The study found no significant association between Gn dosage, Gn duration, and blastocyst euploidy. The mean number of euploidy blastocysts in PPOS protocol was significantly lower than that of GnRH-a protocol and GnRH-ant protocol (0.75 ± 0.92 vs. 1.79 ± 1.78 vs. 1.80 ± 1.67). The euploidy rate per biopsy blastocyst (48.4% vs. 49.1% vs. 33.1%), per oocyte retrieved (15.0% vs. 14.7% vs. 10.5%), and per MII oocyte (17.7% vs. 16.4% vs. 11.7%) were significantly higher in the cycles using the GnRH-ant and GnRH-a protocols than that of PPOS protocol group. Regression analyses indicated that, compared with the PPOS protocol, the GnRH-ant protocol was positively associated with the euploid blastocyst rate and the mean number of euploid blastocysts, whereas the GnRH-a protocol showed no such relationship.

**Limitations and reasons for caution:**

The main limitation of this study was the retrospective design. Although this study also used other tests to account for confounding factors and reduce potential bias, multiple tests have its own weaknesses.

**Conclusions:**

GnRH-ant protocol was the most effective for PGT-A cycles. The findings emphasize the need for personalized treatment strategies, considering patient demographics, and optimizing COS protocols to enhance the chances of successful outcomes in ART procedures.

**Supplementary Information:**

The online version contains supplementary material available at 10.1186/s12958-023-01166-7.

## Introduction

Aneuploidy, characterized by an abnormal number of chromosomes, is a prominent factor contributing to embryo implantation failure and miscarriage in assisted reproductive technology (ART). Research has demonstrated a correlation between advanced maternal age and increased aneuploid prevalence in human pre-implantation embryos [1, 2]. However, even younger individuals exhibit a substantial incidence of embryo aneuploidy, while the euploidy rates in donor egg cycles vary across different fertility clinics [3]. Hence, it is crucial to consider maternal age and the potential impact of iatrogenic factors associated with ART, specifically the controlled ovarian stimulation (COS) process, which may contribute to the occurrence of embryonic chromosomal abnormalities.

COS plays a significant role in ART by generating sufficient oocytes and euploid blastocysts. In certain cases, such as in women of advanced age, recurrent spontaneous abortion (RSA), and recurrent implantation failure (RIF), pre-implantation genetic testing for aneuploidy (PGT-A) can effectively increase the chances of successful transfer of euploid blastocysts in subsequent frozen embryo transfer (FET) cycles.

The three most commonly used COS protocols in ART are the Gonadotropin-Releasing Hormone agonist (GnRH-a) downregulation, Gonadotropin-Releasing Hormone antagonist (GnRH-ant), and progestin-primed ovarian stimulation (PPOS). Several studies have explored which COS protocol yields a higher blastocyst euploidy rate, but findings have been inconsistent [4–6]. Some studies suggested that the early follicular long-acting GnRH-a long protocol (EFLL) was associated with more euploid embryos than the mid-luteal short-acting gonadotropin-releasing hormone (GnRH) agonist long protocol (MLSL) [4]. Conversely, a study indicated that euploidy rates in PGT-A cycles did not differ using the PPOS or GnRH-ant protocol. In contrast, one study reported that the GnRH-ant protocol was associated with a higher rate of aneuploidy than the GnRH-a downregulation protocol [5]. However, no study has compared the euploidy rates in PGT-A cycles with the GnRH-a downregulation, GnRH-ant, or PPOS protocols.

Considering the above, we performed a multicenter retrospective study of PGT-A cycles using the GnRH-a downregulation, GnRH-ant, and PPOS protocols. This study aimed to evaluate whether COS protocol is associated with the blastocyst euploidy rate in PGT-A cycles.

## Materials and methods

### Study design

Data for this retrospective study were obtained from three reproductive medicine centers: the Reproductive Medicine Center of Xiangya Hospital, the Reproductive Medicine Center of the Third Affiliated Hospital of Guangxi Medical University, and Chengdu Xinan Gynecology Hospital. Data on patient demographics, protocols, and embryonic outcomes were collected for the PGT-A cycles performed between January 2019 and August 2022.

### Participants

The study included PGT-A cycles with the GnRH-a, GnRH-ant, or PPOS protocols. PGT-A was primarily conducted for advanced maternal age (AMA), recurrent spontaneous abortion (RSA), and repeated implantation failure (RIF). The baseline demographic parameters, COS parameters, and embryo outcomes were recorded. Next-generation sequencing (NGS) was used to test the chromosomal aneuploidy of the embryos.

### Ovarian stimulation protocols

For patients undergoing the GnRH-a protocol, either long- or short-acting GnRH-a was administered during the mid-luteal phase. After approximately two weeks, the patients were monitored using ultrasound, and serum sex hormone levels were tested. Gonadotropin (Gn) was administered for COS after achieving downregulation criteria, and the initial dose of Gn was determined according to the antral follicle count (AFC), age, anti-mullerian hormone (AMH), and body mass index (BMI). The dosage was adjusted based on the ovarian response. Human chorionic gonadotropin (hCG) trigger 6000-10000IU was administered when ≥ 2 dominant follicles reached ≥ 18 mm.

For the GnRH-ant protocol, the initial Gn level was introduced on day 2 or 3 of the menstrual cycle. The Gn dose was adjusted depending on follicle development with the addition of GnRH-ant (cetrorelix, 0.25 mg/day) when the mean diameter of the dominant follicle reached 14 mm on the trigger day.

In the PPOS protocol, Gn and Progesterone (dydrogesterone, 20 mg/day) were administered on day 2 or 3 of the menstrual cycle. Progesterone was administered orally once daily until trigger day. The prescribed dose ensured pituitary gland suppression, as used in previous studies.

For the above three protocols, the initial dose of FSH was based on female age, AFC, AMH level, and BMI. The ovarian response was monitored using transvaginal ultrasonography and serum sex hormone examinations. Final oocyte maturation was triggered with 6000-10000IU hCG or/and GnRH-a 0.2 mg, followed by oocyte retrieval 36 h later when ≥ 2 dominant follicles reached ≥ 18 mm.

After the cumulus granulosa cells were stripped, the MII oocytes were retrieved and fertilized by intracytoplasmic sperm injection (ICSI) 4 h later. Fertilization was assessed 16–18 h after ICSI. All embryos were cultured to the blastocyst stage in G1/G2 sequential medium in a tri-gas incubator at 37 °C with 6% CO_2_ and 5% O_2_. All blastocysts were evaluated on day 5 or 6 after fertilization according to the Gardner’s scoring system.

### Trophectoderm biopsy and pre-implantation genetic testing for aneuploidy

Trophectoderm biopsies were taken from blastocysts graded ≥ 3 BC on day 5 or 6 after fertilization. Approximately 3–5 trophectoderm cells were aspirated with a biopsy pipette. Whole-genome amplification of the biopsied trophectoderm cells were performed according to the manufacturer’s instructions. Normal or balanced blastocysts were identified by next-generation sequencing (NGS). PGT-A results were recorded as euploid, aneuploid, or mosaic with a segmental aneuploidy resolution of 16 Mb.

### Outcome measures

The primary outcome was the euploid blastocyst rate per biopsied blastocysts. Secondary outcomes were the number of euploid blastocysts per cycle.

### Statistical analysis

Descriptive statistics were used to summarize the baseline variables. Continuous data were described as mean ± SD and compared with one-way analysis of variance (ANOVA). Categorical data were described as numbers and percentages and compared using chi-squared statistics. Linear regression analysis evaluated the association between the COS protocol and the number of euploid blastocysts. The post-hoc test used to demonstrate the mean significant difference between two groups. All statistical analyses were performed using SPSS (version 25.0, Chicago, IL, USA) with a two-sided *P* < 0.05 considered statistically significant.

### Ethics approval

This study was approved by the ethics committees of the Reproductive Medicine Center, Xiangya Hospital, Central South University.

## Results

This study included 457 PGT-A cycles from three reproductive medicine centers, with 152, 126, and 179 cycles performed using the PPOS, GnRH-a, and GnRH-ant protocols, respectively. The baseline characteristics of the three groups are shown in Table 1. No significant differences were observed in female BMI, infertility type, and infertility duration among the PPOS, GnRH-a, and GnRH-ant protocol groups. However, cycles using the PPOS protocol had an older male and female age than the other groups (Female age:37.11 ± 4.48 vs. 33.82 ± 5.25 vs. 34.01 ± 4.88; Male age:39.72 ± 6.11 vs. 35.98 ± 6.48 vs. 36.48 ± 6.59). Accordingly, serum AMH level (2.27 ± 2.33 vs. 3.16 ± 2.08 vs. 3.62 ± 2.37 ng/ml), AFC (10.03 ± 6.72 vs. 11.76 ± 4.633 vs. 12.87 ± 6.30), E2 on trigger day (2400.83 ± 1532.08 vs. 3485.49 ± 2017.96 vs. 3073.83 ± 1689.18 pg/ml) was significantly lower, and basal FSH level (8.15 ± 2.96 vs. 6.83 ± 2.19 vs. 6.96 ± 1.85 IU/L) was significantly higher in PPOS protocol group compared with GnRH-a and GnRH-ant protocol groups. Infertility duration was longer for couples in the PPOS protocol compared to GnRH-a and GnRH-ant protocol groups (3.22 ± 3.38 years vs. 2.98 ± 3.14 years vs. 3.04 ± 3.04 years). Patients treated with the GnRH-a protocol had a higher number of total Gn dosage (2589.83 ± 821.74 vs. 2090.30 ± 650.09 vs. 2024.59 ± 531.60 IU), Gn days (10.95 ± 2.00 vs. 8.93 ± 2.00 vs. 9.17 ± 1.45 days), and a lower LH level on trigger day (1.93 ± 1.33 vs. 3.52 ± 2.39 vs. 3.18 ± 2.32 ng/ml) compared with those with PPOS protocol and GnRH-ant protocol.

**Table 1:** Characteristics of the embryos from PGT-A cycles with three COS protocols

	PPOS protocol	GnRH-a protocol	GnRH-ant protocol	*P* value
Cycles (n)	152	126	179	
Female age(y)	37.11 ± 4.48 ^a^	33.82 ± 5.25 ^b^	34.01 ± 4.88 ^b^	< 0.001
Female BMI (kg/m^2^)	22.33 ± 2.65	22.04 ± 2.63	21.97 ± 2.77	0.453
Male age(y)	39.72 ± 6.11^a^	35.98 ± 6.48 ^b^	36.48 ± 6.59 ^b^	< 0.001
Infertility type				0.899
Primary (n/%)	30 (19.7%)	25 (19.8%)	32 (17.9%)	
Secondary (n/%)	122 (80.3%)	101 (80.2%)	147 (82.1%)	
Infertility duration (y)	3.32 ± 3.38	2.98 ± 3.14	3.04 ± 3.04	0.643
bFSH (IU/L)	8.15 ± 2.96 ^a^	6.83 ± 2.19 ^b^	6.96 ± 1.85 ^b^	< 0.001
AMH (ng/ml)	2.27 ± 2.33 ^a^	3.16 ± 2.08 ^b^	3.62 ± 2.37 ^b^	< 0.001
AFC	10.03 ± 6.72 ^a^	11.76 ± 4.63 ^b^	12.87 ± 6.30 ^b^	< 0.001
Gn days (d)	8.93 ± 2.00 ^b^	10.95 ± 2.00 ^a^	9.17 ± 1.45 ^b^	< 0.001
Gn dosage (IU)	2090.30 ± 650.09 ^b^	2589.83 ± 821.74 ^a^	2024.59 ± 531.60 ^b^	< 0.001
E2 on trigger day (pg/ml)	2400.83 ± 1532.08 ^a^	3485.49 ± 2017.96 ^b^	3073.83 ± 1689.18 ^b^	< 0.001
LH on trigger day (IU/L)	3.52 ± 2.39 ^a^	1.93 ± 1.33 ^b^	3.18 ± 2.32 ^b^	< 0.001

BMI, body mass index; bFSH, basal follicle-stimulating hormone; LH, luteinizing hormone; E2, estradiol; AMH, anti-Müllerian hormone; Gn, gonadotropin

^a.b^ means significant differences between the two groups

The oocyte and blastocyst outcomes of the three groups are shown in Table 2. Patients in the PPOS protocol group showed a significantly lower number of oocytes retrieved (7.16 ± 5.00 vs. 12.22 ± 6.25 vs. 12.04 ± 6.40), MII oocyte (6.40 ± 4.48 vs. 10.96 ± 5.60 vs. 10.22 ± 5.57), 2PN fertilization zygote (4.63 ± 2.99 vs. 8.40 ± 4.68 vs. 7.88 ± 4.81), blastocyst formed (3.36 ± 2.14 vs. 6.71 ± 4.27 vs. 5.96 ± 3.74), blastocyst biopsy (2.26 ± 1.48 vs. 3.79 ± 2.57 vs. 3.76 ± 4.47) compared with GnRH-a protocol and GnRH-ant protocol groups. After NGS testing, the mean number of euploidy blastocysts was significantly lower than that of GnRH-a protocol and GnRH-ant protocol (0.75 ± 0.92 vs. 1.79 ± 1.78 vs. 1.80 ± 1.67). The euploidy rate per biopsy blastocyst (48.4% vs. 49.1% vs. 33.1%), per oocyte retrieved (15.0% vs. 14.7% vs. 10.5%), and per MII oocyte (17.7% vs. 16.4% vs. 11.7%) were significantly higher in the cycles using the GnRH-ant and GnRH-a protocols compared to the PPOS protocol group.

**Table 2:** Oocyte and blastocyst outcomes of PGT-A cycles with three COS protocols

	PPOS protocol	GnRH-a protocol	GnRH-ant protocol	*P* value
Cycles (n)	152	126	179	
No. of oocytes retrieved	7.16 ± 5.00 ^a^	12.22 ± 6.25 ^b^	12.04 ± 6.40 ^b^	< 0.001
No. of MII	6.40 ± 4.48 ^a^	10.96 ± 5.60 ^b^	10.22 ± 5.57 ^b^	< 0.001
MII rate (n/%)	973/1088 (89.43%) ^a^	1381/1540 (89.67%) ^a^	1830/2155 (84.92%) ^b^	< 0.001
No. of fertilized oocytes (2PN)	4.63 ± 2.99 ^a^	8.40 ± 4.68 ^b^	7.88 ± 4.62 ^b^	< 0.001
2PN Fertilization rate (%)	758/973 (77.90%) ^a^	1125/1381 (81.46%) ^a^	1482/1830 (80.98) ^a^	0.073
No. of blastocysts formed	3.36 ± 2.14 ^a^	6.71 ± 4.27 ^b^	5.96 ± 3.74 ^b^	< 0.001
No. of blastocyst biopsied	2.26 ± 1.48 ^a^	3.79 ± 2.57 ^b^	3.76 ± 4.47 ^b^	< 0.001
No. of euploidy blastocyst	0.75 ± 0.92 ^a^	1.79 ± 1.78 ^b^	1.80 ± 1.67 ^b^	< 0.001
Euploidy rate per biopsied blastocyst (n/%)	114/344 (33.14%) ^a^	226/460 (49.13%) ^b^	323/668 (48.35%) ^b^	< 0.001
Euploidy rate per oocyte retrieved (n/%)	114/1088 (10.48%) ^a^	226/1540 (14.68%) ^b^	323/2155 (14.99%) ^b^	< 0.001
Euploidy rate per injected MII oocytes (n/%)	114/973 (11.72%) ^a^	226/1381 (16.36%) ^b^	323/1830 (17.65%) ^b^	< 0.001

MII, mature oocytes; 2PN, two pronuclei

^a.b^ means significant differences between the two groups

Furthermore, linear regression analyses were performed to evaluate the association between COS protocols and the rate of euploidy per blastocyst biopsied and between COS protocols and the mean number of euploid blastocysts. The results indicated that the GnRH-ant protocol was associated with the euploidy rate, female age, and AMH. Regression analyses indicated that, compared with the PPOS protocol, the GnRH-ant protocol was positively associated with the euploid blastocyst rate per biopsied blastocysts, whereas the GnRH-a protocol showed no such relationship (Table 3). Additionally, the analyses revealed that apart from female age and basal FSH, AFC, and E2 on the trigger day, the COS protocol was independently linked to the mean number of euploid blastocysts (Supplementary Table 1).

**Table 3:** Correlation between blastocyst euploidy rate per biopsied blastocyst and COS protocols

Model	Unstandardized coefficients	Standardized coefficients	t	Sig.	Adjusted R^2^
	B	Beta			
Constant	1.42		5.76	0.000	0.311
Protocol_2	0.330	0.098	1.818	0.174	
Protocol_3	0.652	0.214	4.251	0.001	
Female age	− 0.105	− 0.356	− 5.308	0.000	
Female BMI	0.034	0.061	1.413	0.399	
Male age	− 0.001	− 0.007	− 0.105	0.727	
bFSH	− 0.054	− 0.090	− 2.020	0.114	
AMH	− 0.008	− 0.012	− 0.227	0.015	
AFC	− 0.037	− 0.140	− 2.639	0.547	
E2 on trigger day	0.000	0.279	5.079	0.500	
LH on trigger day	− 0.021	− 0.031	− 0.693	0.475	
Gn dosage	0.000	− 0.058	− 0.786	0.775	
Gn days	0.056	0.075	1.028	0.948	

The COS protocol was set as a dummy variable, and the PPOS protocol was selected as the reference group

Protocol_2 refers to the GnRH-a protocol, and Protocol _3 refers to the GnRH-ant protocol

## Discussion

The present study included 457 PGT-A cycles with 915 biopsied blastocysts from three reproductive centers and compared the results of blastocyst euploidy. Our results indicated that the GnRH-ant protocol was the most effective for PGT-A cycles. To our knowledge, this is the first study to analyze and compare blastocyst euploidy among the three commonly used COS protocols.

The results of a previous study showed that the euploidy rates of embryos formed from donor young donor eggs varied among reproductive centers [3]. Recently, another study revealed varying euploidy rates among physicians within a single center due to physician-specific COS protocols for donors [7]. Therefore, it is essential to choose an appropriate COS protocol for PGT-A to ensure that at least one euploid embryo is obtained. Numerous studies have compared COS protocols in terms of clinical pregnancy or live birth outcomes and have arrived at different conclusions. Despite these studies, the COS protocol that can achieve better embryo euploidy remains unclear.

Our study found that the PPOS protocol cycle had significantly lower numbers of euploid blastocysts and lower euploidy rates than those using the GnRH-a and GnRH-ant protocols. We noted a negative association between the GnRH-ant protocol and the mean number of euploid blastocysts with regression analysis while controlling for female age, BMI, male age, basal FSH, AMH, AFC, total Gn dosage, Gn days, E2, and LH levels on trigger days.

The PPOS protocol with orally administered progestins is beneficial economically compared to GnRH analogs. The higher patient comfort and lower cost of progesterone compared with GnRH make the PPOS protocol attractive for COS. However, we found a lower euploid rate regardless of whether the blastocyst was biopsied or per oocyte retrieved, even though the PPOS protocol had a higher MII rate than the GnRH-ant protocol and had fertilization rates similar to those of the GnRH-a and GnRH-ant protocols. Similar to our study, an RCT of oocyte donation cycles showed that lower biochemical pregnancy rate, clinical pregnancy rate, and live birth rate in the PPOS protocol group than in the the GnRH-ant protocol, although both groups obtained similar numbers of mature oocytes in oocyte donation cycles [8].

In contrast to our study, Marca et al. (2019) found that blastocysts and euploid blastocysts count per patient and the number of euploid embryos per injected oocyte were similar between the PPOS and GnRH-ant protocol [9]. Another study found that the euploidy rate per embryo biopsy was similar in patients undergoing the PPOS or GnRH-ant protocols [6]. While most studies have indicated that elevated progesterone levels on trigger day do not negatively affect the euploid rate and the outcome of FET [10–12], some reports have suggested that elevated progesterone levels negatively affect embryo development [13, 14]. However, blastulation time has been associated with euploidy [15, 16].

The GnRH-a and GnRH-ant protocols are well-established methods for treating COS. Current reports comparing the clinical outcomes of these two protocols based on prior meta-analyses and systematic reviews are inconsistent [17, 18]. Clinical outcomes were mainly limited to clinical pregnancy, ongoing pregnancy, live births, and birth defects. However, only one study has compared the effects of GnRH-a and GnRH-ant on embryonic aneuploidy. The results showed that the GnRH-ant protocol is associated with a higher aneuploidy rate in early aborted tissues and blastocysts compared to the GnRH-a long protocol [5].

Contrary to this study, we found a similar but increasing trend in the euploidy rate per blastocyst biopsied, per oocyte retrieved, and per MII oocyte in the GnRH-ant protocol. This discrepancy may be due to the inclusion of aborted tissues after IVF/ICSI-ET and PGT, including PGT-SR and PGT-A in the Wang et al. study, whereas our study included only patients who underwent PGT-A. Compared to the GnRH-a protocol, the GnRH-ant protocol had a shorter COS duration and lower Gn dosage. Therefore, the GnRH-ant protocol provides greater time and economic benefits.

Although COS aims to recruit several follicles for an increased likelihood of obtaining an euploid embryo that results in a healthy conceptus, several studies have suggested that high Gn stimulation might be embryotoxic and/or increase aneuploidy rates by enhancing the abnormal segregation of chromosomes during meiosis [19, 20]. Another study showed that the duration of the ovarian stimulation treatment was correlated with the aneuploidy rate [4, 21]. Our study found no significant association between Gn dosage, Gn duration, and blastocyst euploidy. Similarly, a retrospective cohort study with 2230 IVF/PGT-A cycles indicated that the Gn dosage, duration of ovarian stimulation, E2 level, follicle size at ovulation trigger, and the number of oocytes retrieved within certain ranges did not appear to significantly influence euploidy rates [22].

To our knowledge, this is the first study to investigate the association between three commonly used COS protocols and blastocyst euploidy. Originality, multiple centers, and relatively large sample sizes could be considered points of strength. The main limitation of our study was its retrospective design. Although additional testing was used to account for confounders and reduce potential bias in our population, there are weaknesses in the use of multiple testing. Moreover, the trigger reagents were not analyzed, and triggers with GnRH-a were used in partial cycles with the PPOS and GnRH-ant protocols. However, GnRH-a triggers are associated with higher euploidy rates in hyper responders [23].

Our study demonstrated that the GnRH-ant is the preferred COS protocol for PGT-A treatment. Further well-designed prospective studies with large sample sizes are required to validate our findings and provide more conclusive evidence for selecting a COS protocol for PGT-A treatment.

### Electronic supplementary material

Below is the link to the electronic supplementary material.

**Supplementary Material 1:** Supplementary Table 1 Correlation between number of euploidy blastocyst and COS protocols.

## Data Availability

The data included in this study will be shared on the request to the corresponding author.
