# Supplementary material for: Correlation between controlled ovarian stimulation protocols and euploid blastocyst rate in pre-implantation genetic testing for aneuploidy cycles
Source: Reprod Biol Endocrinol. 2023 Dec 6;21:118. doi: 10.1186/s12958-023-01166-7 (PMC10698966; doi:10.1186/s12958-023-01166-7)
Supplement: Supplementary file 1 — Supplementary Material 1: Supplementary Table 1 Correlation between number of euploidy blastocyst and COS protocols. [file 12958_2023_1166_MOESM1_ESM.docx]

| Supplemental Table 1. Correlation between the number of euploidy blastocyst and COS protocols | | | | | |
| --- | --- | --- | --- | --- | --- |
| Model | Unstandardized Coefficients | Standardized Coefficients | t | Sig. | Adjusted R^2^ |
|  | B | Beta |  |  |  |
| Constant | 3.96 |  | 4.82 | 0.000 | 0.324 |
| Protocol_2 | .330 | .098 | 1.818 | .070 |  |
| Protocol_3 | .652 | .214 | 4.251 | .000 |  |
| Female age | -.105 | -.356 | -5.308 | .000 |  |
| Female BMI | .034 | .061 | 1.413 | .158 |  |
| Male age | -.001 | -.007 | -.105 | .916 |  |
| bFSH | -.054 | -.090 | -2.020 | .044 |  |
| AMH | -.008 | -.012 | -.227 | .820 |  |
| AFC | -.037 | -.140 | -2.639 | .009 |  |
| E2 on trigger day | .000 | .279 | 5.079 | .000 |  |
| LH on trigger day | -.021 | -.031 | -.693 | .489 |  |
| Gn dosage | .000 | -.058 | -.786 | .432 |  |
| Gn days | 0.056 | 0.075 | 1.028 | .305 |  |

The COS protocol was set as a dummy variable, and the PPOS protocol was selected as the reference group.

Protocol_2 refers to the GnRH-a protocol, and Protocol _3 refers to the GnRH-ant protocol.
